# Proper Hepatic Artery Pseudoaneurysm and Dissection: A Rare Case of Segmental Arterial Mediolysis

**DOI:** 10.7759/cureus.89894

**Published:** 2025-08-12

**Authors:** Christina Lee, Kei Yamada, Fredric Gordon

**Affiliations:** 1 Gastroenterology, Tufts Medical Center, Boston, USA; 2 Interventional Radiology, Tufts Medical Center, Boston, USA; 3 Transplant Hepatology, Tufts Medical Center, Boston, USA

**Keywords:** abdominal pain, adult gastroenterology, elevated liver enzyme, hepatic artery pseudoaneurysm, liver dysfunctions, segmental arterial mediolysis (sam)

## Abstract

Segmental arterial mediolysis (SAM) is a rare vasculopathy of unknown etiology. Due to the rarity of SAM and limited literature on the disease, there is a lack of consensus on the diagnostic criteria and therapeutic management. Thus, patients can present with serious complications of SAM, which include aneurysm, dissection, occlusion, stenosis, hemorrhage, and infarction. We report a rare case of a patient diagnosed with SAM within the liver, as well as unravel the diagnostic approaches and therapeutic management of SAM in this patient’s case.

## Introduction

Segmental arterial mediolysis (SAM) is a rare non-atherosclerotic, non-inflammatory vasculopathy of unknown etiology affecting medium to large-sized arteries [1]. SAM typically presents with abdominal pain, and serious complications of SAM include aneurysm, dissection, occlusion, stenosis, hemorrhage, and infarction. Due to limited literature on the disease, there is a lack of consensus on the diagnostic criteria and therapeutic management [1]. Computed tomography (CT) followed by angiography was the most common modality utilized for diagnostic imaging. Typical imaging features include the following: “string of beads” appearance from the alternations between aneurysmal dilations and stenoses, thrombosis, and dissection, all of which were seen in our case [2,3]. Subsequent endovascular interventions, specifically coiling of aneurysmal vessels, were used for the majority of cases [1,4]. We report a rare presentation of a patient diagnosed with SAM within the liver.

## Case presentation

Patient is a 67-year-old female individual, with a past medical history of hypertension and gastroesophageal reflux disease (GERD), who presented with one day of sudden-onset crampy, diffuse abdominal pain associated with nausea and non-bloody emesis. Her review of systems was notable for a pre-syncopal episode prior to her hospital visit. She denied use of alcohol, intravenous drugs, and herbal products.

On admission, the following were her vital signs: blood pressure 150/95 mm Hg, heart rate 74 beats per minute, respiratory rate 23 breaths per minute, and oxygen saturation of 96% on room air. Physical examination was notable for a distended abdomen with diffuse tenderness to palpation. The following were her notable laboratory test results: white blood cell count 14 K/μL, hemoglobin 10.4 g/dL, creatinine 1.58 mg/dL (baseline creatinine 0.8 mg/dL), ferritin 4,242 ng/mL, C-reactive protein 184 mg/L (Table 1). Her liver function tests showed aspartate transaminase 6,249 U/L, alanine transaminase 3,205 U/L, alkaline phosphatase 220 U/L, total bilirubin 0.3 mg/dL, albumin 3.8 g/dL, and international normalized ratio 0.9 (Table 1). Infectious workup, including *Treponema pallidum* antibodies and human immunodeficiency virus, was unremarkable. Rheumatologic workup was negative for the following: anti-nuclear antibody, anti-neutrophil cytoplasmic antibodies, anti-myeloperoxidase antibodies, anti-smooth muscle antibody, liver-kidney microsomal type 1 antibody, and complement proteins. Thus, other vasculitides and anti-neutrophil cytoplasmic antibody-associated vasculitis, such as microscopic polyangiitis, were excluded from the differential. Further laboratory tests revealed negative hepatitis A, B, and C serologies, normal ceruloplasmin and alpha-1 anti-trypsin levels, and absence of hereditary hemochromatosis mutation. Thus, acute hepatitis, Wilson's disease, alpha-1 antitrypsin deficiency, and hereditary hemochromatosis were not in the differential for this patient.

**Table 1:** Patients' laboratory tests

Laboratory tests	Patients' laboratory results	Reference ranges
White blood cell count	14 K/μL	4-10 K/uL
Hemoglobin	10.4 g/dL	11.8-14.8 g/dL
Creatinine	1.58 mg/dL	0.55-1.30 mg/dL
Ferritin	4,242 ng/mL	10-307 ng/mL
C-reactive protein	184 mg/L	0-4.99 mg/L
Aspartate transaminase	6,249 U/L	6-42 U/L
Alanine transaminase	3,205 U/L	0-55 U/L
Alkaline phosphatase	220 U/L	30-130 U/L
Total bilirubin	0.3 mg/dL	0.2-1.2 mg/dL
Albumin	3.8 g/dL	3.2-5.0 g/dL
International normalized ratio	0.9	0.9-1.1

**Figure 1:**
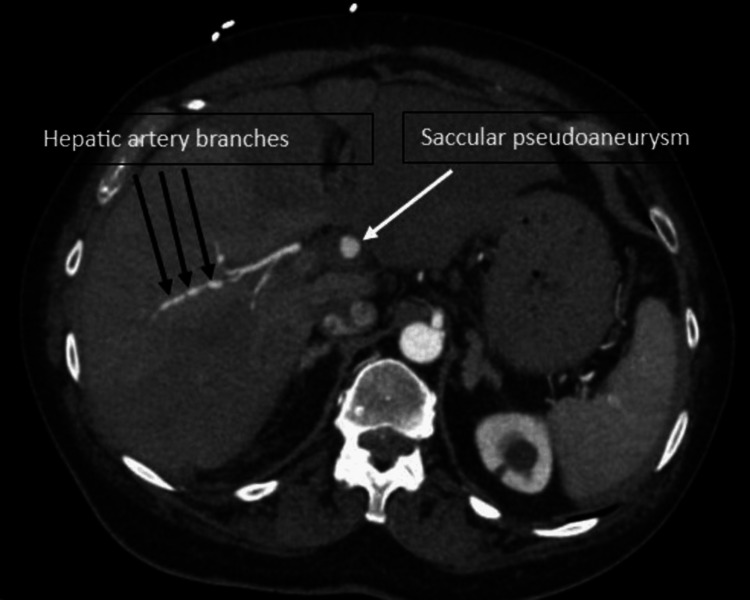
Proper hepatic artery pseudoaneurysm Axial contrast-enhanced CT shows saccular pseudoaneurysm of the proper hepatic artery (white arrow) and the bead-like irregularities of the hepatic artery branches (black arrows).

An axial contrast-enhanced CT of the abdomen showed a 1.5 cm saccular pseudoaneurysm arising from the proper hepatic artery, diffuse irregularity of the hepatic artery branches, moderate volume hemoperitoneum, and probable left hepatic artery thrombosis (Figure 1).

**Figure 2:**
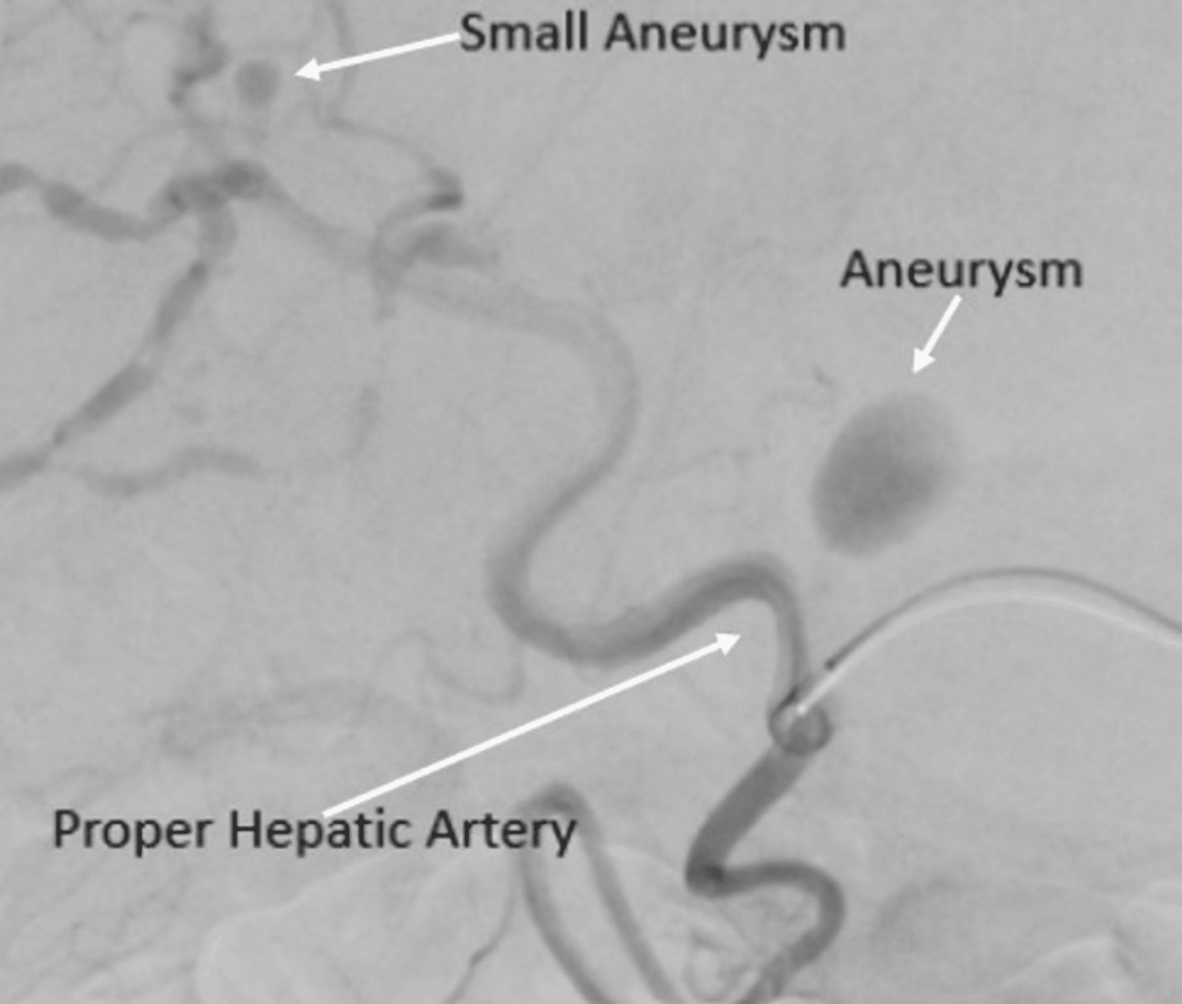
Pre-embolization of the proper hepatic artery pseudoaneurysm Celiac artery angiogram shows the proper hepatic artery pseudoaneurysm as well as one of many other small aneurysms throughout the liver.

A celiac artery angiogram confirmed the pseudoaneurysm of the proper hepatic artery with multiple other small aneurysms throughout the liver (Figure 2).

**Figure 3:**
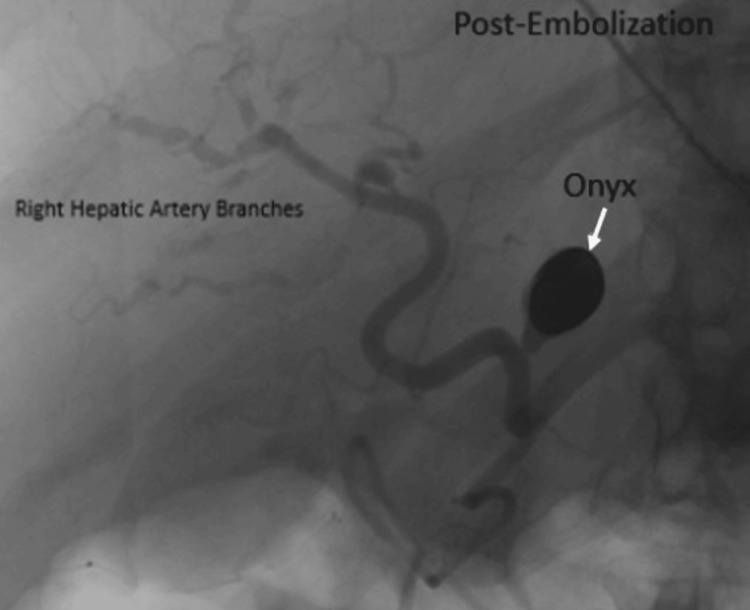
Post-embolization of the proper hepatic artery pseudoaneurysm Celiac artery angiogram shows the proper hepatic artery after embolization with Onyx.

Subsequently, the patient underwent treatment with Onyx 18 (ethylene vinyl copolymer; Medtronic, Minneapolis, MN) embolization of the proper hepatic artery pseudoaneurysm (Figure 3).

There were no post-procedure complications. Subsequent computer tomography angiography of the head, neck, and chest was obtained to determine the distribution of affected vessels; reassuringly, no aneurysms or dissections were noted. Post-procedure laboratory test results demonstrated improving inflammatory markers and transaminases within two weeks after discharge.

## Discussion

SAM usually affects the medium to large mesenteric arteries (53%) followed by the hepatic (45%), celiac (36%), renal (26%), and splenic arteries (25%) [1]. Our case was unique in that only the hepatic arterial branches were involved.

SAM is a non-atherosclerotic, non-inflammatory disease [1]; however, other connective tissue diseases, such as Type IV Ehlers-Danlos, and vasculitides, such as ANCA-associated vasculitis and giant cell arteritis, can mimic this disease. Patient demographics, clinical features, distribution of affected vessels, laboratory findings, histology, and imaging characteristics are all utilized to differentiate SAM from other mimicking diseases. Typical histological features of SAM are the following: outer layer of media predominantly affected, alternating stenoses and aneurysms with disruption of elastin [4]. Typical imaging features include the following: “string of beads” appearance from the alternations between aneurysmal dilations and stenoses, thrombosis, and dissection, all of which were seen in our case [2,3].

The diagnosis of SAM in our patient was confirmed with the clinical presentation, laboratory tests, and the radiological findings on her CT and angiography.

In our case, subsequent C-reactive protein improved during her hospital course without any steroids, suggesting that her initial presentation of hemoperitoneum from the proper hepatic artery pseudoaneurysm dissection was the likely source of inflammatory marker elevation. Normalization of inflammatory markers and transaminases after the embolization also leads to further confirmation.

For diagnosis, CT followed by angiography was the most common modality utilized [1,4]. Early detection of SAM with imaging can mitigate the serious complications of the disease. SAM without clinical manifestations does not need treatment [1]. However, patients presenting with life-threatening complications, such as hemodynamic instability or end-organ damage/ischemia, require endovascular therapies, as seen in our patient case [5].

## Conclusions

This patient's demographics, clinical features, distribution of affected vessels, laboratory findings, and imaging characteristics were all utilized to differentiate SAM from other mimicking diseases; thus, we trust that this case report will bring clinical value to patients presenting in a similar fashion. Specifically, this case sheds light on the importance of prompt diagnostic imaging, specifically CT angiography, followed by prompt endovascular intervention for patients with hemodynamic and/or clinical instability. Due to the lack of current literature on this rare disease, little is known about it. More in-depth research will be essential in improving our understanding and knowledge about the course of the disease, in order to develop a safe diagnostic and therapeutic consensus for SAM.

## References

[1] Skeik N, Olson SL, Hari G, Pavia ML (2019). Segmental arterial mediolysis (SAM): systematic review and analysis of 143 cases. Vasc Med.

[2] Baker-LePain JC, Stone DH, Mattis AN, Nakamura MC, Fye KH (2010). Clinical diagnosis of segmental arterial mediolysis: differentiation from vasculitis and other mimics. Arthritis Care Res (Hoboken).

[3] Alhalabi K, Menias C, Hines R, Mamoun I, Naidu S (2017). Imaging and clinical findings in segmental arterial mediolysis (SAM). Abdom Radiol (NY).

[4] Shenouda M, Riga C, Naji Y, Renton S (2014). Segmental arterial mediolysis: a systematic review of 85 cases. Ann Vasc Surg.

[5] Chatterjee T, Stephens J, Roy M (2020). Segmental arterial mediolysis: an under-recognized cause of chronic abdominal pain. Eur J Case Rep Intern Med.

